# Congenital Microphthalmia with Intraorbital Cyst: A Rare Case Report

**DOI:** 10.1155/2019/3640175

**Published:** 2019-12-11

**Authors:** Bishow Raj Timalsina, Gulshan Bahadur Shrestha, Madhu Thapa

**Affiliations:** Department of Ophthalmology, B.P. Koirala Lions Centre for Ophthalmic Studies, Kathmandu, Nepal

## Abstract

Microphthalmia is considered to be the most common congenital malformation of the eye after congenital cataract. However, its association with intraorbital cyst is considered to be very rare. Most of the lesions are still misdiagnosed as orbital tumor and teratomas as there is a general paucity of data reported in literature. Herein, we report a rare case of congenital microphthalmia with intraorbital cyst in an eight-month-old male patient.

## 1. Introduction

Congenital microphthalmia with intraorbital cyst is a rare condition occurring due to defective closure of the embryonic fissure as well as over-growth of the inner layer of the optic cup [1]. The fissures begin to close at about 11 mm stage and is completed by 18 mm stage. As the inner layer of the optic cup develops faster, the margins of the fissures becomes slightly everted. Presence of a fully formed retina at the margins of the fissure prevents the closure of the optic cup leading to the formation of a typical coloboma without overlying the retina [2]. Microphthalmia is characterized by a small but recognizable eye with the eye elements such as the lens, choroid, and retina [3]. In contrast to this, anophthalmia is characterized by the complete absence of the eye due to the lack of development or arrest of differentiation of the optic vesicles in early stage of development [4]. The prevalence rate of microphthalmia is found to be 1.4–3.5 per 10,000 births [5]. Microphthalmia with orbital cyst is considered to be extremely rare with no agreed prevalence rate [1].

Cysts associated with microphthalmia and anophthalmia represent two points on the spectrum of the colobomatous eye disorder and may create difficulty in clinical distinction [5].

## 2. Case Report

An eight-month-old male was presented by the parents with the chief complaints of inability to open the right eye since birth and gradual progressive swelling underneath the right lower lid for three months.

The past medical history revealed that the patient was taken to the local eye hospital one week after birth with the chief complaints of inability to open the right eye (RE) since birth. The medical reports revealed that the right globe was not visible and the left eye (LE) was normal with no developmental anomalies at that time. The USG A+B scan showed chorioretinal coloboma with cystic (hyperechoic) space behind the chorioretinal surface and a small optic nerve stump on the RE with normal LE findings.

**Figure 1:**
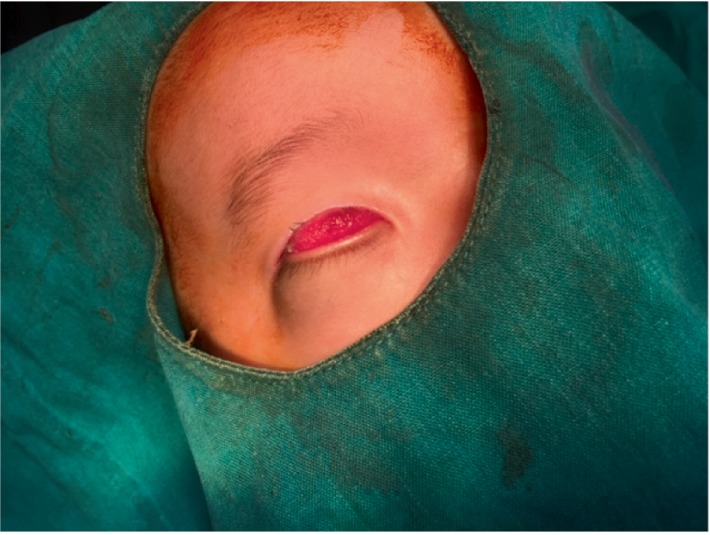
Preoperative view of right eye.

Three months later, cystic swelling appeared from the inferior orbit that was gradually increasing in size and was more prominent during coughing. This lead to the right lower lid ectropion and exposure of the palpebral conjunctiva associated with redness and occasional discharge. Swelling gradually increased in size with mild keratinization of the exposed surface (Figure 1). The LE showed inferior key hole iris (coloboma) with chorioretinal coloboma on indirect ophthalmoscopy. The USG A+B scan showed no identifiable RE structures while LE showed chorioretinal coloboma.

**Figure 2:**
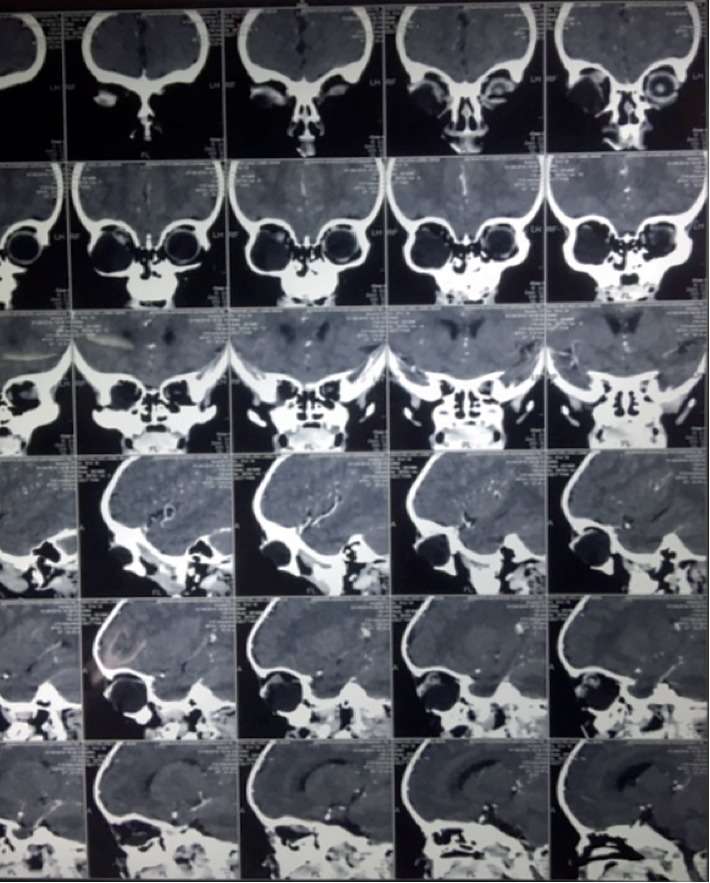
MDCT showing cyst and microphthalmic eye.

**Figure 3:**
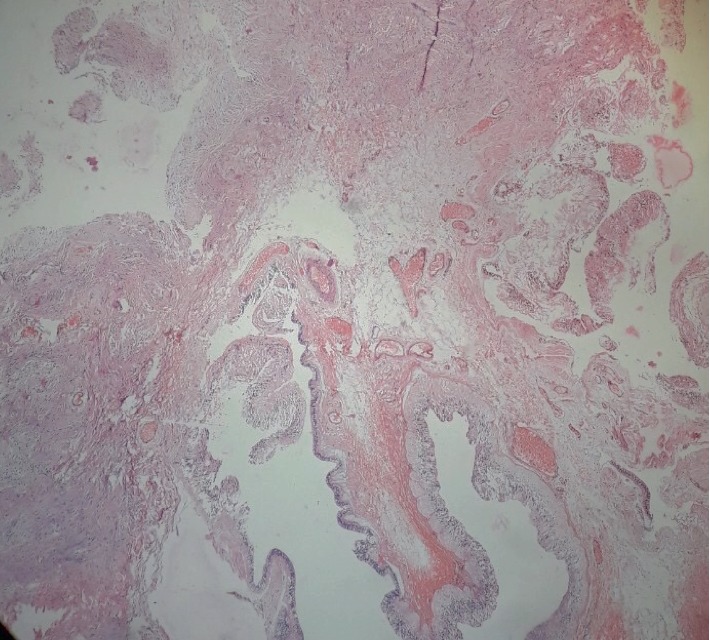
Glial tissue with cystic component 200×.

**Figure 4:**
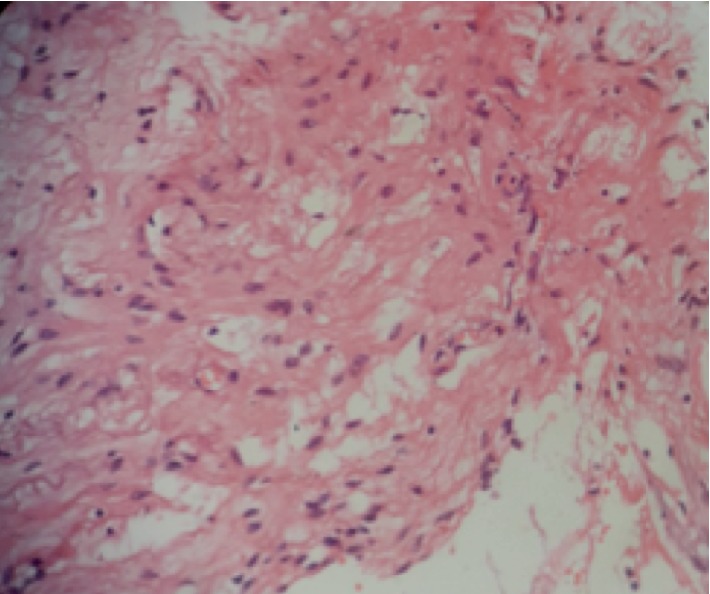
Higher power view of Glial tissue 400×.

At eight months, when the child was brought to our center, multidetector computed tomography (MDCT) was advised which showed non enhancing right intraorbital cyst with small right globe and dysplastic optic nerve (Figure 2). There was no intracranial communication or extension. Findings were suggestive of colobomatous cyst. Right orbitotomy with right eye enucleation and cyst excision was performed under general anesthesia and the specimen was sent for histopathological examination. Histopathology revealed heterotopic glial tissue with cystic component in the orbit consistent with choriostomatous cyst (Figures 3 and 4). The eyeball was found to be atrophic. Based on these findings, the diagnosis of the right eye microphthalmos with intraorbital cyst and left eye complete coloboma was confirmed.

## 3. Discussion

Microphthalmia is considered to be the most common congenital malformation of the eye after congenital cataract. Its association with the intraorbital cyst is considered as a rare developmental anomaly of the globe that can affect one or both the eyes [6]. Studies have shown that unilateral cases are more common than bilateral cases. Out of 150 cases reported in literature, only one third of them were bilateral [7].

The detection of microphthalmia can be done in early neonatal period. It typically presents as a protruding mass in the inferior orbit associated with microphthalmic eye. The globe may be completely surrounded by the cyst in some cases, while others may present with very rudimentary displaced microphthalmic eye, thus creating difficulty in identifying the eye clinically as in our case [2].****In some cases, cysts associated with microphthalmia can be diagnosed clinically, whereas in others, it can be doubtful. Studies have shown that additional imaging can be valuable in these cases. Orbital ultrasound A+B scan is of great importance in identifying orbital cysts [5]. In our case too, orbital ultrasound A+B scan along with MDCT proved to be valuable in diagnosing the condition. These imaging modalities are not only useful in diagnosis, but can also be very helpful in identifying other abnormalities such as of the brain. Systemic defects such as the cleft lip, basal encephalocele, mid brain deformity, microcephalus, agenesis of corpus callosum, and saddle nose have been found to be associated with the condition mostly with bilateral cases. In such cases, preoperative imaging could be of great importance [4]. In our case, it was an isolated malformation without any systemic defects.

Although it is clearly a defined entity, sometimes it becomes difficult to differentiate it from some lesions such as congenital cystic eye, meningocele, arachnoid cyst, primary optic nerve sheath cysts, and teratomas of the orbit. Lieb et al. has mentioned the importance of imaging techniques in ruling out these differentials [2].

Histopathologically, cyst in a microphthalmic eye is similar to the congenital cystic eye. Nevertheless in congenital cystic globes, the cyst is located centrally or slightly upward in the orbit and histologically it lacks normal ocular structures and shows the presence of the cyst lined with neuroglial tissue. However, in micropthalmos with cyst, there is bulging of the lower eyelid as the cyst is attached to the inferior portion of the globe along with evidence of ocular development in conjunction with a small cornea, iris, ciliary body, lens, vitreous cavity, retina, and choroid [6].

The treatment strategy for patients with microphthalmos and orbital cyst depends on several factors such as visual potential, age at presentation, and volume of the orbital content [5]. Clinical assessment of the orbital volume can be done by Duke-Elder classification [8]. Patients with poor orbital volume, if treated early with implant and conformers can show good cosmetic results. Studies have shown that enucleation during childhood compromises the orbital growth especially when no orbital implant is used [9, 10].Not only that, small cyst helps to stimulate orbital expansion much more effectively than an artificial implant [5]. However, individualized interventions are utmost to improve esthetic outcomes [11]. Chaudhry et al. have proposed the treatment protocol for congenital microphthalmos and orbital cyst. In case of mild microphthalmia, if the cyst is small, cyst aspiration, and observation is recommended, whereas if it is large affecting cosmesis, excision of the cyst should be performed. In case of severe microphthalmia, both the cyst and globe should be excised with replacement of volume [6]. In our case, as the cyst was large and rudimentary microphthalmic eye was presented, enucleation, and cyst excision was used as the treatment option. Repeated follow up should be kept in consideration to prevent recurrence and development of neoplasia [7].

## 4. Conclusion

In this case report, we tried to report a rare case of congenital microphthalmia with intraorbital cyst. Several studies are still required to understand the spectrum of this complex condition, which is considered as one of the cause for childhood blindness. Advanced ultrasound technology using three dimensional image and computed tomography can be helpful in early diagnosis of the lesion which in turn provide great help in counseling the parents and provide appropriate treatment options in early neonatal life.
